# Inherited disorders of intermediary metabolism – a group-based approach

**DOI:** 10.1515/medgen-2021-2053

**Published:** 2021-05-14

**Authors:** Johannes Zschocke

**Affiliations:** Institute of Human Genetics, Medical University Innsbruck, Peter-Mayr-Str. 1, 6020Innsbruck, Austria

**Keywords:** inherited metabolic diseases, intermediary metabolism, classification, ICIMD

## Abstract

In the recently developed International Classification of Inherited Metabolic Disorders (ICIMD), more than one third of the 1450 listed conditions involve gene products required for intermediary metabolism. 225 of these diseases represent deficiencies of enzymes or transport proteins in the breakdown of nutrients, many of which cause acute “metabolic” presentations with typical biochemical features that are amenable to specific treatments. A group-based approach to these conditions not only assists in understanding and remembering them but facilitates the best choice of diagnostic tests and acute treatment. This review describes the basic characteristics of the 25 disease groups in the four categories of nutrient breakdown in intermediary metabolism, outlines the often relatively straight-forward diagnostic approach, and summarizes important therapeutic principles. It should also assist in the retrospective identification of likely metabolic disorders in the family history for genetic counselling.

## Introduction

The recently developed International Classification of Inherited Metabolic Disorders (ICIMD) [1] contains a total number of 1450 individual disorders. They are defined as primary disturbances of a biochemical pathway with a known (or assumed) primary genetic cause, irrespective of the clinical consequences. Most conditions are caused by reduced-function mutations in single genes although some are due to activating mutations or other genetic or genomic alterations, e. g. in the mitochondrial genome. Within the ICIMD the disorders are hierarchically structured into 24 categories comprising 124 disease groups (Table 1). The first four categories refer to the disorders of nutrient breakdown in intermediary metabolism and comprise disorders of the metabolism of amino acids, peptides/amines, carbohydrates, and fatty acids/carnitine/ketones. The disorders of intermediary energy metabolism include a large number of diverse conditions that interfere mostly with mitochondrial oxidative phosphorylation. Two categories are classified as “other” disorders of intermediary metabolism; one of them covers the small but growing number of disorders of metabolite repair. This category comprises deficiencies of enzymes that do not have a function in a particular biochemical pathway but remove metabolites generated through non-specific side reactions of other enzymes. The ICIMD has been endorsed by all international societies for inherited metabolic disorders and is due to be used for the Orphanet classification and different electronic database resources. Its hierarchical structure can also serve as a basis for didactic purposes and textbooks [2]. Here we review the prototypical clinical and biochemical presentations as well as main diagnostic and therapeutic approaches to the different disorders of nutrient breakdown in intermediary metabolism. This category contains many of the well-known inherited metabolic diseases that share elements of the clinical presentation and are recognized by standard “metabolic analyses.” The discussion of the clinical approach to other metabolic disease groups is beyond the scope of this manuscript.

**Table 1:** Disease categories in the International Classification of Inherited Metabolic Disorders.

Disorders of intermediary metabolism: nutrients	Disorders of amino acid metabolism
	Disorders of peptide and amine metabolism
	Disorders of carbohydrate metabolism
	Disorders of fatty acid and ketone body metabolism
Disorder of intermediary metabolism: energy	Disorders of energy substrate metabolism
	Mitochondrial DNA-related disorders
	Nuclear-encoded disorders of oxidative phosphorylation
	Disorders of mitochondrial cofactor biosynthesis
	Disorders of mitochondrial DNA maintenance and replication
	Disorders of mitochondrial gene expression
	Other disorders of mitochondrial function
Disorders of intermediary metabolism: others	Disorders of metabolite repair/proofreading
	Miscellaneous disorders of intermediary metabolism
Disorders of lipid metabolism and transport	Disorders of lipid metabolism
	Disorders of lipoprotein metabolism
Disorders of heterocyclic compounds	Disorders of nucleobase, nucleotide, and nucleic acid metabolism
	Disorders of tetrapyrrole metabolism
Disorders of complex molecule and organelle metabolism	Congenital disorders of glycosylation
	Disorders of organelle biogenesis, dynamics, and interactions
	Disorders of complex molecule degradation
Disorders of cofactor and mineral metabolism	Disorders of vitamin and cofactor metabolism
	Disorders of trace elements and metals
Disorders of metabolic cell signalling	Neurotransmitter disorders
	Endocrine metabolic disorders

## Simplified overview of intermediary metabolism

**Figure 1:** Intermediary metabolism: overview and diagnostic tests.

**Figure 2:**
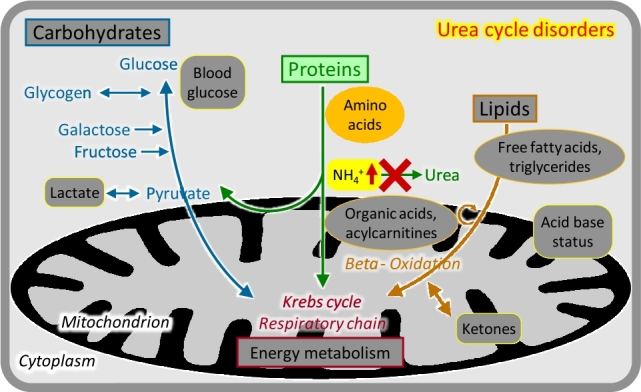
Urea cycle disorders.

**Figure 3:**
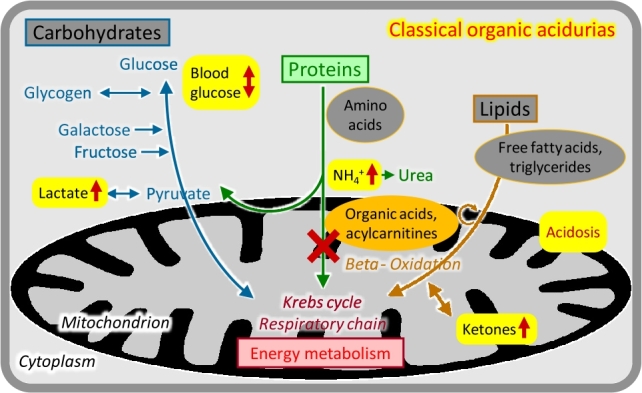
Classical organic acidurias.

**Figure 4:**
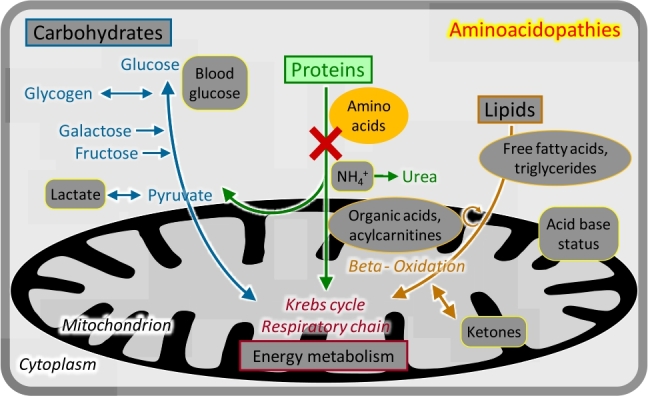
Aminoacidopathies.

**Figure 5:**
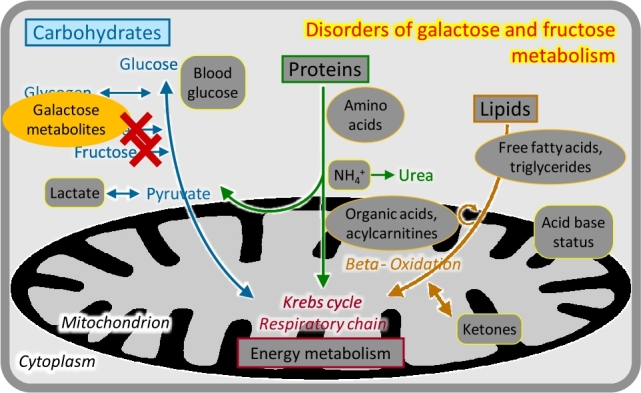
Disorders of galactose and fructose metabolism.

**Figure 6:**
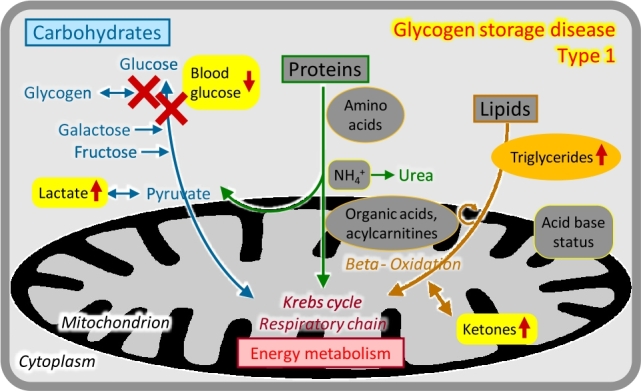
Glycogen storage disease type 1.

**Figure 7:**
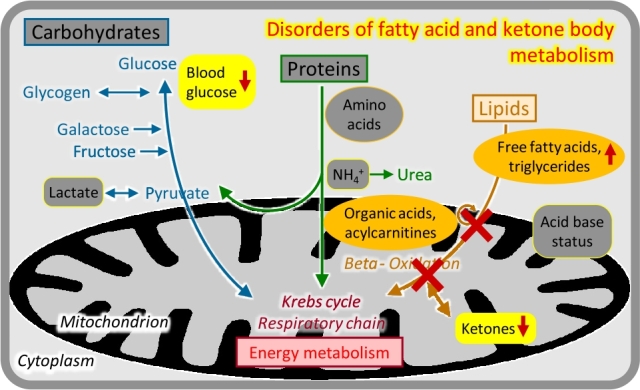
Disorders of fatty acid and ketone body metabolism.

Different biochemical categories and laboratory analyses are highlighted in different colours. Yellow fields indicate basic laboratory tests that should be performed in every patient with an acute illness potentially due to in inherited metabolic disease. Orange fields special metabolic investigations that may identify specific metabolic changes. In the subsequent Figures 2–7, fields that are shaded dark grey are not of primary relevance for the diagnosis of diseases in the respective group.

Breakdown of the three major nutrient components – carbohydrates, proteins/peptides, and lipids – ultimately results in oxidative phosphorylation in the mitochondria for the generation of adenosine triphosphate (ATP). The main elements relevant for clinical needs and the relevant biochemical tests are depicted – very simplified – in Figure 1.

– Cytosolic breakdown of glucose leads to pyruvate, which can be reversibly converted to lactate, the end product of anaerobic glycolysis. After transport into the mitochondria, pyruvate is either fed into the Krebs cycle via the pyruvate dehydrogenase complex, or serves as precursor of various metabolites via pyruvate carboxylase. Glycogen, the main storage form of glucose, is required for rapid provision of chemical energy, e. g. in the muscle or in the first hours of fasting. The other two main nutrient hexoses – galactose and fructose – are fed into the glucose pathways through specific enzymes.
– Amino acids released from breakdown of proteins and peptides are often modified in the cytosol before removal of the amino group. Deamination results in the generation of organic acids that are further catabolized and finally enter the mitochondrial Krebs cycle. Free ammonium ions (${\mathrm{NH}}_{4}^{+}$) or amino groups are detoxified mostly in the urea cycle.
– Lipids in the form of triglycerides are the main long-term storage compounds for nutritive energy. Their release is started after a few hours of fasting. Coenzyme A (CoA)-activated long-chain fatty acids are transported via the carnitine shuttle into the mitochondria, where they are broken down through successive beta-oxidation cycles. This process generates large amounts of acetyl-CoA, which enters the Krebs cycle or – in the liver – is converted into ketone bodies that function as energy source, e. g. for the brain.

The different nutrient breakdown pathways can be assessed with some *basic laboratory tests* which should be available for the investigation of patients with acute presentations in every hospital: blood glucose, ketones (urine stix test; quantification in blood is more reliable but available only in some centres), acid base status, lactate, and ammonium (in blood). Serum triglyceride levels may provide information on lipolysis although the analysis of free fatty acids is preferable for exact quantification. *Selective metabolic screening tests* that may show specific abnormalities diagnostic for particular disorders of intermediary metabolism include amino acid analysis in plasma/serum, urinary organic acid analysis, and the quantification of acylcarnitines (carnitine esters of mitochondrial CoA compounds) in dried blood spots. These laboratory tests in combination with the individual clinical presentation may allow a rapid diagnosis and start of specific treatment in patients with an acute “metabolic” presentation.

## Disorders of amino acid metabolism

Deficiencies of enzymes involved in amino acid metabolism frequently result in the accumulation of toxic substances leading to acute or chronic organ damage. The brain, liver, and kidneys are most frequently affected. Development of acute symptoms in many diseases is often linked to increased breakdown of protein derived from endogenous catabolism, e. g. during an illness. Rapid diagnosis in these cases may be essential for effective emergency treatment. Other disorders of amino acid metabolism cause chronic, often progressive organ dysfunction. Treatment strategies include (a) reversion and avoidance of protein catabolic states in diseases with acute presentations; (b) specific detoxification measures as required; (c) controlled protein intake with specific reduction of the precursor amino acid in the affected pathway; and (d) supplementation of necessary other amino acids, vitamins, minerals, and trace elements. Most conditions are asymptomatic at birth, and many are recognized in expanded newborn screening programs.

### Urea cycle disorders and inherited hyperammonaemias

Free ammonium derived mostly from amino acid breakdown is a highly neurotoxic metabolite and requires rapid and effective removal. This is achieved by producing water-soluble urea from carbamoyl phosphate (from ammonium and bicarbonate) and the amino group of aspartate in the four-enzyme urea cycle. Depending on the functional severity, disorders that primarily affect ammonium detoxification present with acute or chronic-fluctuating brain dysfunction. In the newborn period, severely affected children after a brief symptom-free interval develop a rapidly progressive encephalopathy with lethargy, hyperventilation, seizures, and coma. Manifestation patterns later in life include acute/episodic or chronic neurologic symptoms. Essential for diagnosis is the determination of blood ammonium in the acute situation (Figure 2). Plasma amino acid analysis usually shows elevated glutamine concentrations and may reveal specific alterations of other amino acids. Treatment includes controlled protein intake, maintenance of an anabolic state, removal of ammonium if necessary by haemodiafiltration, and amino group removal with specific drugs [3]. The most frequent and important urea cycle disorder is *ornithine transcarbamylase* (*OTC*) *deficiency*, which is inherited as an X-linked trait and not only causes mostly severe neonatal hyperammonaemia in boys but may trigger acute lethal metabolic decompensation in previously healthy heterozygous females at any age.

### Organic acidurias

The group of organic acidurias comprises inherited disorders in the metabolism of CoA-activated carboxylic acids mostly derived from deamination of amino acids [4], [5]. The enzymes involved are usually localized in the mitochondria, and the biochemical disturbance often interferes with energy metabolism. As stated in the name, specific organic acidurias are recognized by urinary organic acid analysis but they also generate specific acylcarnitine patterns (Figure 3). Most of the *classical organic acidurias* – such as propionic, methylmalonic, and isovaleric aciduria – are caused by enzyme deficiencies in branched-chain amino acid (valine, leucine, and isoleucine) breakdown, and typically manifest as acute or intermittent encephalopathy sometimes associated with other organ damage (e. g. heart, kidney). Basic metabolic tests often show metabolic acidosis with elevated concentrations of ketones, lactate, and ammonium; hypoglycaemia may occur. No systemic metabolic derangement is usually found in the *cerebral organic acidurias* (e. g. glutaric aciduria) which may show specific neurological symptoms and neuroradiological alterations. Some genetically and biochemically defined organic acidurias can remain asymptomatic throughout life.

### Aminoacidopathies

Breakdown of the 20 proteinogenic amino acids requires a large number of specific enzymes. The initial steps are mostly localized in the cytosol. Deficiencies of these enzymes cause clinical manifestations usually depending on the specific toxicity of the accumulating metabolites. Diagnosis is based on the analysis of plasma amino acids (Figure 4); urinary amino acid analysis is not relevant for the majority of conditions.

– Most disorders of *branched-chain amino acid* metabolism are classified as organic acidurias; the main exception is maple syrup urine disease, which causes neonatal or late-onset encephalopathy.
– The disorders of *phenylalanine and tyrosine* metabolism include phenylketonuria (PKU, untreated progressive intellectual disability), alkaptonuria (generation of dark pigments, osteoarthritis), and three types of tyrosinaemia. Accumulating metabolites in tyrosinaemia type 1 are highly toxic; their production is prevented by pharmaceutical blockage of an upstream enzyme with the drug nitisinone (NTBC).
– Disorders of the metabolism of *sulphur-containing amino acids* (*methionine, homocysteine, cysteine*) *and hydrogen sulphide* cause predominately neurological symptoms. *S*-adenosylmethionin is the most important methyl group donor in cellular metabolism, and its replenishment requires remethylation of homocysteine to methionine linked to the folate cycle. Classical homocystinuria causes a Marfan-like disease.
– Disorders of *serine* metabolism are multi-system disorders, in severe cases with prenatal onset (Neu–Laxova syndrome). *Non-ketotic hyperglycinaemia* caused by the mitochondrial glycine cleavage system is an important epileptic encephalopathy.
– Disorders affecting the *other amino acids* (ornithine, proline, and hydroxyproline; lysine, hydroxylysine, and tryptophan; glutamate/glutamine and aspartate/asparagine; and histidine) are mostly rare and cause a wide range of neurological and non-neurological manifestations. Some conditions (such as histidinaemia) remain asymptomatic. Alanine – the amino acid corresponding to pyruvate – is often elevated in mitochondrial disorders.
– The disorders of *amino acid transport* include cystinuria, a renal reabsorption deficiency of lysine, arginine, ornithine, and cysteine, responsible for 6–8 % of renal stones in childhood.

## Disorders of peptide and amine metabolism

Four disease groups – disorders of the metabolism of glutathione, other peptides, methylamines, and polyamines – are classified as disorders of peptide and amine metabolism. Diagnosis usually requires special biochemical tests or molecular genetic analyses. Most of the conditions are rare; a notable exception is *trimethylaminuria* or fish odour syndrome, which is characterized by unpleasant fish-like body odour exacerbated by the intake of choline-containing foods or carnitine [6]. An attenuated form linked to homozygosity for a common variant allele of the *FMO3* gene is predicted to affect up to 4 % of Europeans [7].

## Disorders of carbohydrate metabolism

### Disorders of galactose and fructose metabolism

There are two main nutritive disaccharides: lactose (milk sugar, galactose-glucose disaccharide) and sucrose (table sugar, glucose-fructose disaccharide). Utilization of galactose (Gal) and fructose (Fru) involves phosphorylation at the carbon-1 position; the respective metabolites Gal-1-P and Fru-1-P are toxic particularly for the liver and kidneys. Correspondingly, deficiencies of the enzymes required for Gal-1-P and Fru-1-P processing cause progressive liver and renal dysfunction starting with the intake of the respective disaccharide [8]. Classical *galactosaemia* becomes manifest with milk feeds after birth and is also an important cause of cataracts; it is detected by analyses of galactose metabolites (Figure 5) and enzyme studies in newborn screening programs. *Hereditary fructose intolerance* becomes manifest after weaning or upon addition of sucrose/fructose to the diet; there is no specific biochemical analysis, and the diagnosis is made by mutation analysis. Both conditions are treated by removal of the respective carbohydrate from the diet.

### Disorders of glycogen metabolism (glycogen storage diseases [GSDs], glycogenoses)

Glycogen, the main storage form of glucose in humans, is particularly abundant in the liver – where it is used for glucose homeostasis – and the muscle – where it is used for the provision of energy during exercise [9], [10]. Enzymes required for glycogen synthesis or release may be ubiquitous or may have two (liver- or muscle-specific) isoforms. Correspondingly, diseases in this group have three prototypic clinical presentations:

– Liver glycogenoses typically present with hypoglycaemia at the onset of fasting (e. g. 3–4 hours after meals), hepatomegaly and other storage features, growth retardation, and variable other features. The most frequent disease is *GSD type 1* (*von Gierke*). Laboratory analyses in the acute phase show marked lipaemia and elevated triglycerides caused by massive lipolysis, elevated lactate and uric acid, and ketonuria (Figure 6). Treatment is based on frequent meals, slowly resorbed carbohydrates, and continuous nasogastric tube feeding overnight.
– Muscle glycogenoses frequently cause exercise intolerance, muscle cramps, and myoglobinuria; treatment is mostly symptomatic. *GSD type 2* (*Pompe*) is characterized by severe muscle weakness and cardiomyopathy, which can be treated with enzyme replacement therapy.
– Mixed/generalized glycogenoses show both liver and muscle manifestations including cardiomyopathy.

Diagnosis in all glycogen storage disorders is based on molecular genetic analyses in conjunction with enzyme studies.

### Other disorders of carbohydrate metabolism

Group-specific clinical presentations based on the function in intermediary metabolism can also be defined for the other disorders of carbohydrate metabolism:

– Disorders of gluconeogenesis show recurrent hypoglycaemia, elevated lactate, and ketosis. Fructose-1,6-bisphosphatase deficiency, which is close to Glc-6-P in gluconeogenesis, also has marked hepatomegaly and may resemble GSD type 1. Progressive neurodegeneration and severe lactic acidosis are features of enzyme deficiencies closer to the Krebs cycle, such as in pyruvate carboxylase deficiency.
– Disorders of glycolysis can cause haemolytic anaemia combined with variable neurologic, muscle, and retinal manifestations.
– The disorders of pentose metabolism include *glucose-6-phosphate dehydrogenase deficiency*, an X-chromosomal disease with recurrent haemolysis triggered by specific foods (favism), drugs, and other factors, prevalent in malaria regions.
– Disorders of carbohydrate transmembrane transport and absorption cause a variety of renal, gastrointestinal, neurological, or multi-system manifestations, based on the deficient transport functions.

## Disorders of fatty acid and ketone body metabolism

Enzyme deficiencies in the mitochondrial import and oxidation of CoA-activated fatty acids, or in the production of ketone bodies, have variable but overlapping clinical presentations that are typically related to catabolic states, e. g. after 8–12 hours of fasting [11]. Urinary organic acid analysis and acylcarnitine analysis in dried blood spots may show specific abnormalities (Figure 7).

– Insufficient ketogenesis in conjunction with inhibition of gluconeogenesis causes potentially life-threatening acute hypoketotic hypoglycaemic coma, often in the second half of the first year of life. This is the typical presentation of medium-chain acyl-CoA dehydrogenase deficiency, the most frequent inherited fatty acid oxidation disorder which is now fortunately included in most newborn screening programs.
– Severe deficiency of the carnitine shuttle and long-chain fatty acid oxidation enzymes can present like a respiratory chain defect in the neonatal period, with progressive coma, cardiomyopathy, liver failure, severe lactic acidosis, and multi-system organ failure.
– Attenuated variants of long-chain fatty acid oxidation and the carnitine shuttle often show adolescent or adult onset chronic muscle weakness, pain, recurrent rhabdomyolysis, and/or cardiomyopathy.

Specific treatment involves the avoidance of fasting, reversion of catabolic states, and supplementation of medium-chain fatty acids and/or carnitine in some conditions.

## Conclusion

Many disorders of nutrient breakdown in intermediary metabolism are relatively easy to diagnose based on the clinical presentation in relation to metabolic states, and on the results of a few basic laboratory tests combined with selective metabolic screening analyses. This is essential for rapid implementation of effective specific treatments which are available for many conditions. The exact diagnosis is usually confirmed by molecular genetic analyses.
